# Supplementary material for: Asymmetric dynamics of DNA entering and exiting a strongly confining nanopore
Source: Nat Commun. 2017 Aug 30;8:380. doi: 10.1038/s41467-017-00423-9 (PMC5577289; doi:10.1038/s41467-017-00423-9)
Supplement: Supplementary file 1 — Supplementary Information [file 41467_2017_423_MOESM1_ESM.pdf]

Description of Supplementary Files

File Name: Supplementary Information
Description: Supplementary Figures, Supplementary Notes, Supplementary Table and Supplementary References

File Name: Peer Review File

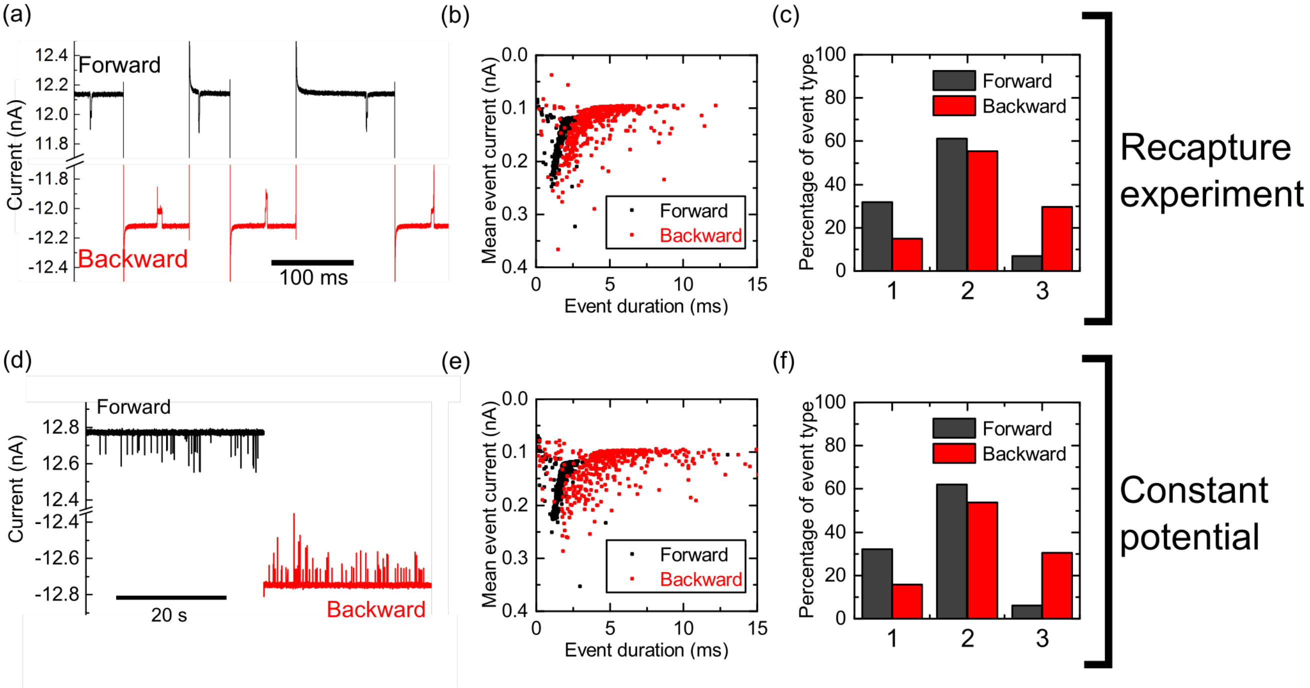

Supplementary Figure 1. Comparison of translocation dynamics when using ping-pong (recapture) experiments and when using constant potential. (a)-(c) contain the data shown in Figs. 1 and 2 in the main text and were made with data from recapture experiments. (d) shows data recorded with the same nanopore using constant potential rather than switching. The slight change in ionic current level is attributed to a small amount of evaporation from the nanopore fluidics. (e) and (f) show the corresponding statistics in the case of constant potential which mirror those measured by recapture experiments.

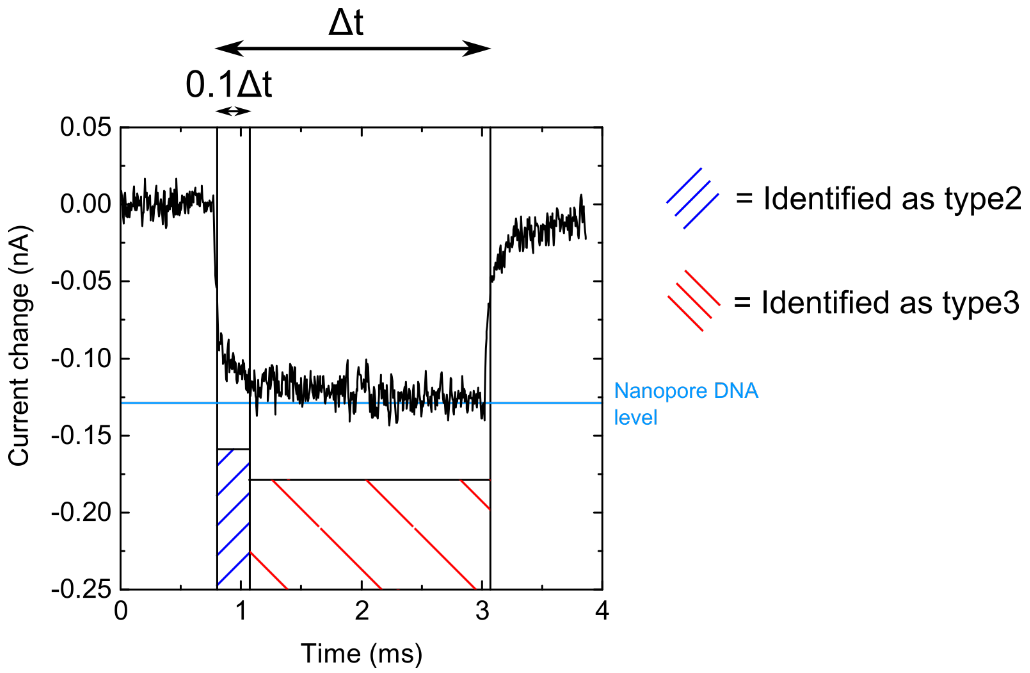

Supplementary Figure 2. Figure demonstrating the definitions of the event types. A type 2 event is designated by an event where the ionic current crosses 30 pA below the nanopore DNA level (the average current blockade from all translocations in the data set) in the first tenth of the translocation time - indicated by the blue box. A type 3 event does not cross the type 2 threshold area but deviates more than 50 pA from the nanopore DNA level during the remainder of the event as indicated by the red box. A type 1 event (as shown in this trace) does not cross either threshold. The beginning and end of the event is set as 60 pA from the baseline.

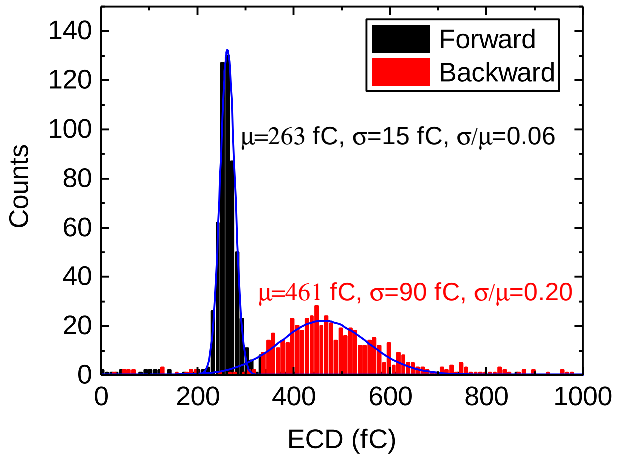

Supplementary Figure 3. Comparison of distribution of event charge deficit values for forward and backward directions. The data is the same as presented in Figure 2a in the main text. The values show the parameters of Gaussian functions fitted to the distributions.

Supplementary Note 1 - Determination of intra-event peak positions

The workflow of intra-event peak position determination for the DNA ruler is based on that first demonstrated by Bell *et al* [1]. Firstly we plot the event charge deficit (ECD) for all translocations as shown in Supplementary Figure 4a. Fragments of DNA are observed as a tail of low values of ECD and are a consequence of the synthesis procedure [2]. These fragments are filtered out and subsequently folded type 2 translocations are also filtered out as in Supplementary Figure 2. A separate peak finding algorithm is then used to search for intra-event peak positions. Supplementary Figure 4b shows a histogram quantifying the number of peaks detected. 88% of translocation are identified as six peaks for the forward translocation whereas only 57% are measured as six peaks in the backward due to the higher propensity for type 3 events. Only events with six peaks are used for the analysis in Figure 4c in the main text.

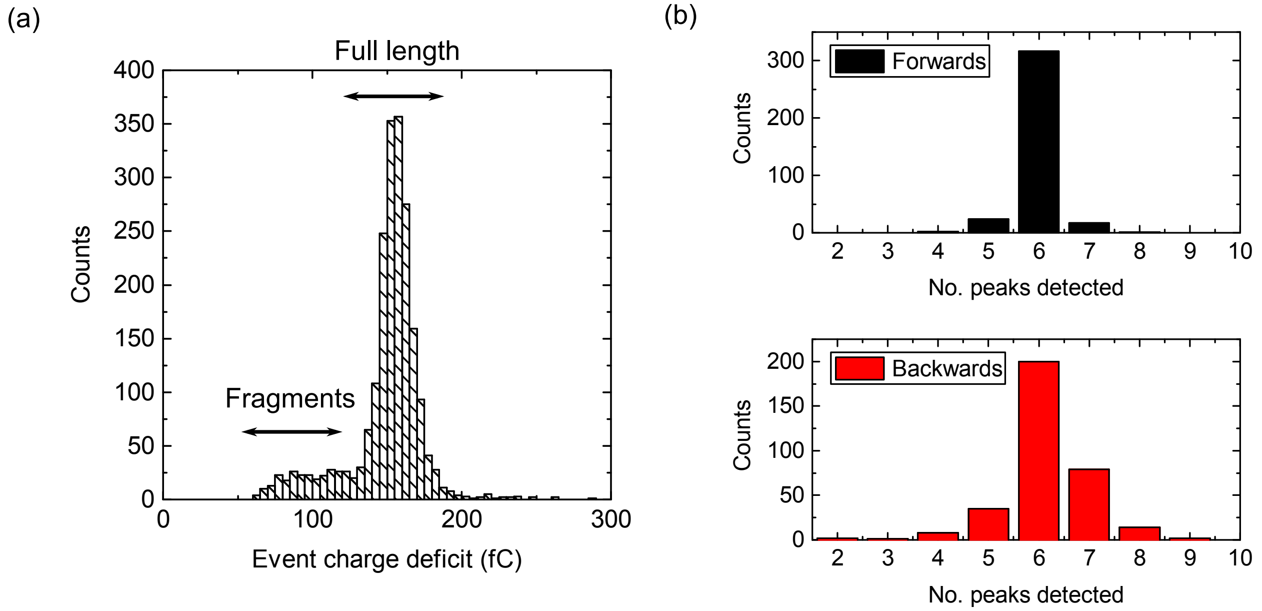

Supplementary Figure 4. (a) ECD histogram of DNA translocations of DNA ruler in forward direction. (b) Histograms of numbers of peaks detection for unfolded, full length molecules in forward and backward directions.

Supplementary Note 2 - mathematical details of the hydrodynamic model

Supplementary Table 1. Values of experimental parameters.

Experimental Parameter	Unit	Value
applied voltage (V)	mV	600
pore radius (R_0)	nm	7
cone semi-angle (α)	radian	0.05
buffer conductivity (σ)	S/m	17
viscosity of electrolyte (μ)	Pa.s	0.0017
permittivity of water (ϵ)	nF/m	0.708
dna radius (a)	nm	1.0
dna length ($L_{\max}$)	nm	2458
debye length (κ^{-1})	nm	0.2

Electric field geometry

At the high ionic concentrations in the experiment, the Debye length is thin compared to other relevent length scales (Supplementary Table 1). Furthermore, due to the reduction of effective charge by Li^+ ions [3], characteristic zeta potentials are ~ 1 mV; much less than the thermal scale $kT/e \sim 33$ mV. Thus, the Dukhin number, which is a measure of the ratio of surface to bulk conductivity, is $Du \sim 10^{-3} \ll 1$, so that surface conductance effects may be neglected and the medium may be regarded as one of uniform conductivity, σ. The local electric field $E(x)$ is therefore radial and may be related to the open pore current (I) using Ohm's law:

$$\sigma E(x) x^2 \delta\Omega = I. \tag{1}$$

Here $\delta\Omega$ is the solid angle of the conical pore and x is the distance measured from the vertex of the cone. The solid angle can be related to the semi-angle of the cone, $\alpha \approx 3^\circ = 0.05$ radians as $\delta\Omega \approx \pi\alpha^2$ and thus,

$$E(x) = E_0 \frac{x_0^2}{x^2} = E_0 \frac{R_0^2}{\alpha^2 x^2} \tag{2}$$

where $x_0 = R_0/\alpha$ defines the position of the nanopore entrance (Supplementary Figure 5),

$$E_0 = \frac{I}{\pi\sigma R_0^2}, \tag{3}$$

and R_0 is the radius at the smallest opening of the pore. The electric force on the part of the DNA inside the nanopore is much greater than that on the part outside. Indeed, outside the nanopore, the field is also radial but the solid angle $\delta\Omega = 4\pi - \pi\alpha^2$. Thus, at the same distance from the origin

$$\frac{E_{\text{out}}(x)}{E_{\text{in}}(x)} \sim \frac{\alpha^2}{4 - \alpha^2} \sim 0.0006 \tag{4}$$

where $E_{\text{out}}(x)$ and $E_{\text{in}}(x)$ are the fields in the reservoir and in the nanopore at an identical distance x from the virtual origin. Therefore we are justified in assuming that only the electric field inside the nanopore is significant.

Derivation of the translocation velocity

The electrophoretic speed of the DNA may be calculated by modelling the fluid flow by the Stokes equation and imposing force balance on the DNA as described in the main text

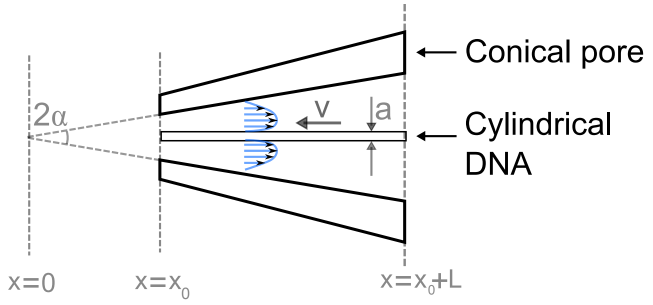

Supplementary Figure 5. Schematic of modelled geometry of a charged rod moving at velocity v through a conical nanopore. The blue arrows indicate the counter propagating electroosmotic flow.

assuming that the DNA moves as a rigid straight rod along the axis of the nanopore - see Supplementaray Figure 5 for the modelled geometry.

As discussed in the main text, in the limit of thin Debye layers, the solution to Stokes equation in the fluid between the DNA and the cylinder in the lubrication limit is

$$u(r,x) = -\frac{\epsilon\zeta_s E(x)}{\mu} + \left\{ v + \frac{\epsilon(\zeta_s - \zeta_p)}{\mu} E(x) \right\} \ln\left(\frac{r}{\alpha x}\right) \Big/ \ln\left(\frac{a}{\alpha x}\right), \tag{5}$$

where $u(r,x)$ satisfies the Helmholtz-Smoluchowski slip boundary conditions at the nanopore wall and on the DNA surface:

$$u(r = \alpha x, x) = -\frac{\epsilon\zeta_s E(x)}{\mu}, \tag{6}$$

$$u(r = a, x) = v - \frac{\epsilon\zeta_p E(x)}{\mu}. \tag{7}$$

The parameters in this equation are, the solution permittivity (ϵ), the zeta potential of the substrate (ζ_s), the zeta potential of the DNA (ζ_p), and $E(x)$ is the local electric field determined from the condition of current continuity on account of our assumption of thin Debye layers. Here the local electric field $E(x)$ is given by Supplementary Equation (2). The force per unit length of the DNA is then

$$f(x) = 2\pi a\mu \left.\frac{du}{dr}\right|_{r=a} = -\frac{2\pi\mu(v + v_e)}{\ln(\alpha x/a)} \tag{8}$$

where

$$v_e = -\frac{\epsilon\Delta\zeta E}{\mu} = -\frac{\epsilon\Delta\zeta E_0}{\mu}\left(\frac{x_0}{x}\right)^2 \equiv v_{e0}\left(\frac{x_0}{x}\right)^2 \tag{9}$$

with $\Delta\zeta = \zeta_p - \zeta_s$. The electrophoretic speed v is now obtained by setting the total force

to zero:

$$F = \int_{x_0}^{x_0+L} f(x)\, dx = -2\pi\mu \int_{x_0}^{x_0+L} \frac{(v+v_e)}{\ln(\alpha x/a)}\, dx$$
$$= -2\pi\mu v \int_{x_0}^{x_0+L} \frac{dx}{\ln(\alpha x/a)} - 2\pi\mu v_{e0} \int_{x_0}^{\infty} \frac{x_0^2}{x^2 \ln(\alpha x/a)}\, dx = 0 \tag{10}$$

where we have replaced the upper integration limit in the latter integral by ∞ as the integrand decreases sufficiently fast to yield a finite value for the integral. This term represents the resultant of the electric driving force on the DNA and the viscous drag of the oppositely streaming adjacent counterion cloud (see later section for an alternative derivation).

The first of the two integrals in Supplementary Equation (10) may be expressed in terms of the logarithmic integral

$$\mathrm{li(x)} = \int_0^{\mathrm{x}} \frac{\mathrm{dt}}{\ln \mathrm{t}}. \tag{11}$$

Thus,

$$F = -\frac{2\pi\mu v a}{\alpha}\left[\mathrm{li}\left(\frac{\alpha \mathrm{L}}{\mathrm{a}} + \frac{\mathrm{R_0}}{\mathrm{a}}\right) - \mathrm{li}\left(\frac{\mathrm{R_0}}{\mathrm{a}}\right)\right] + \lambda_e\, V \tag{12}$$

where

$$\lambda_e = \frac{2\pi\epsilon(\Delta\zeta)R_0}{a}\mathcal{L}\left(\frac{R_0}{a}\right) \tag{13}$$

and

$$\mathcal{L}(x) = \int_x^{\infty} \frac{d\xi}{\xi^2 \ln \xi}. \tag{14}$$

Here λ_e is the effective DNA charge refered to in the main text.

The velocity v may be obtained from the condition of zero net force ($F = 0$) on the DNA:

$$v = \frac{\alpha\lambda_e\, V}{2\pi\mu a}\left[\mathrm{li}\left(\frac{\alpha \mathrm{L}}{\mathrm{a}} + \frac{\mathrm{R_0}}{\mathrm{a}}\right) - \mathrm{li}\left(\frac{\mathrm{R_0}}{\mathrm{a}}\right)\right]^{-1}. \tag{15}$$

We have assumed low potentials and thin Debye layers in deriving Supplementary Equation (15), however, these conditions can be relaxed. In fact, since the Helmholtz-Smoluchowski slip conditions Supplementary Equations (6) and (7) are independent of the Debye-Hückel approximation, the restriction to low potentials is not necessary as long as the Dukhin number remains small. If low potentials can however be assumed, then Supplementary Equation (15) can be shown to be valid for arbitrary Debye length (see later section for alternative derivation).

Backward Translocation

In backward translocation (i.e. escaping confinement), the length of DNA contributing to viscous drag is the actual length of DNA within the conical nanopore as discussed in the main text. Since $v = dL/dt$, where $L(t)$ is the length of DNA residing within the capillary at time t, we have from Supplementary Equation (15)

$$\lambda_e\, V - \frac{2\pi\mu a}{\alpha}\frac{dL}{dt}\left[\mathrm{li}\left(\frac{\alpha \mathrm{L}}{\mathrm{a}} + \frac{\mathrm{R}_0}{\mathrm{a}}\right) - \mathrm{li}\left(\frac{\mathrm{R}_0}{\mathrm{a}}\right)\right] = 0, \tag{16}$$

which may be integrated to obtain the total translocation time T_{back}:

$$T_{\mathrm{back}} = \frac{2\pi\mu a^2}{\alpha^2 V\, \lambda_e}\int_0^{\alpha L_{\max}/a}\left[\mathrm{li}\left(\xi + \frac{\mathrm{R}_0}{\mathrm{a}}\right) - \mathrm{li}\left(\frac{\mathrm{R}_0}{\mathrm{a}}\right)\right]\, d\xi \tag{17}$$

In the main text we numerically integrate equation Supplementary Equation (17) to give the model values shown in Figure 6 (using the integral function of MATLAB). The various parameters that enter into the integral calculated for Figure 6 in the main text are the mean values determined by electron microscopy and force measurements. In Supplementary Figure 6 we illustrate how the model predictions from Equation Supplementary Equation (17) vary according to the measured variation in nanopore geometry. The plots are calculated with the same parameters as Table 1 in the main text but using semi-cone angles at ± one standard deviation from the mean and radii ± one standard deviation from the mean to show how the model predictions vary with these parameters. In general smaller values of semi-cone angle and pore radius decrease the velocity since the closer proximity of the nanopore walls to the DNA increases viscous drag.

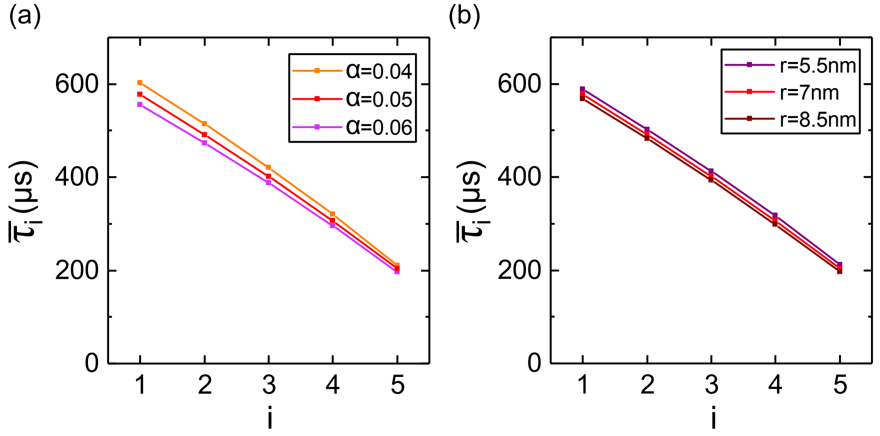

Supplementary Figure 6. (a) Model predictions of intra-event translocation times for semi-cone angles of 0.04 rad, 0.05 rad and 0.06 rad using Supplementary Equation (17) and with all other parameters kept the same as given in Table 1 in the main text. (b) Model predictions of intra-event translocation times for pore radii of 5.5 nm, 7 nm, 8.5 nm using Supplementary Equation (17) and with all other parameters kept the same as given in Table 1 in the main text.

An approximate analytical evaluation of the integral in Supplementary Equation (17) becomes possible if we note that most of the contribution to the integral arises from large values of ξ. Thus, we may use the large argument asymptotic form [4] for the logarithmic integral $\mathrm{li(x)} \sim \mathrm{x}/\ln \mathrm{x}$ to approximate the integrand as follows

$$\mathrm{li}\left(\xi + \frac{\mathrm{R_0}}{\mathrm{a}}\right) - \mathrm{li}\left(\frac{\mathrm{R_0}}{\mathrm{a}}\right) = \frac{\xi + \mathrm{R_0/a}}{\ln(\xi + \mathrm{R_0/a})} - \frac{\mathrm{R_0/a}}{\ln(\mathrm{R_0/a})} \approx \frac{\xi}{\ln(\alpha \mathrm{L_{max}}/2\mathrm{a})}. \tag{18}$$

Since the logarithmic function varies slowly we have replaced it by its value at $\xi = \xi_{\max}/2$, where $\xi_{\max} = \alpha L_{\max}/a$ is the upper limit of integration. On substituting in Supplementary Equation (17) and evaluating the integral, we get

$$T_{\text{back}} = \frac{\pi \mu a^2}{\alpha^2 V \ \lambda_e} \frac{(\alpha L_{\max}/a)^2}{\ln(\alpha L_{\max}/2a)}. \tag{19}$$

The form Supplementary Equation (19) is however useful as it shows how the translocation time (T_{back}) scales with the total length of the DNA ($L_{\max}$). As discussed in the main text, if we ignore the logarithmic factor, we find an approximate scaling $T_{\text{back}} \sim L_{\max}^2$, and a more accurate "scaling exponent" may be obtained by logarithmically differentiating Supplementary Equation (19);

$$n \equiv \frac{d(\ln T_{\text{back}})}{d(\ln L_{\max})} = 2 - \frac{1}{\ln(\alpha L_{\max}/2a)}. \tag{20}$$

DNA buckling theory

The theory of buckling of a beam under a compressive load was first considered by Euler for point forces but a full solution for a beam with a distributed load was provided by Greenhill [5]. In the present problem, we have a distributed load given by the function $f(x)$ in Supplementary Equation (8) which consists of an electric component $\sim 1/(x^2 \ln x)$ and a purely viscous component $1/\ln x$. The problem of determining the critical length, H, of DNA beyond which it would buckle under the applied distributed load $f(x)$ may be formulated as an eigenvalue problem and solved numerically. Here we only seek an estimate, so we will simplify the problem by replacing the distributed force $f(x)$ by two equal and opposite point forces (P) representing the electric and viscous effects. The former is located at the nanopore tip and the latter at a distance $\ell_B/2$, where ℓ_B is the largest length of DNA within the nanopore that is stable against buckling. The critical condition for buckling is then [5]

$$P = \frac{\beta EI}{(\ell_B/2)^2}, \tag{21}$$

where E is the Young's modulus, I the area moment of inertia and β is a dimensionless number. If we use Euler's point force model, then $\beta = \pi^2/4 \approx 2.5$ but if we regard the viscous force to be distributed uniformly over the length ℓ_B, as assumed by Greenhill, then $\beta \approx 2$.

For DNA, it is more convenient to express EI in terms of the persistence length ℓ_p. This is done by equating the bending energy of a beam of length and radius of curvature $\sim \ell_p$ to kT:

$$\frac{EI}{2\ell_p} = kT \tag{22}$$

Using Supplementary Equation (22) in Supplementary Equation (21) we have

$$\frac{\ell_B}{\ell_p} = \left(\frac{8\beta kT}{\ell_p F_{tether}}\right)^{1/2}. \tag{23}$$

The quantity ℓ_B is a new length scale for DNA mechanics that we will call the buckling length. Using l_p= 30 nm, T=296 K and F_{tether}=3.4 pN, we have $\ell_B/\ell_P \sim 0.8$. That is, the buckling length is of the same order as the persistence length.

Derivation of Supplementary Equation (12) in the case of arbitrary Debye length

We consider Stokes flow $\mathbf{u} = \hat{\mathbf{x}} u(x,r)$ in the annulus between the DNA translating with a velocity v and the inner surface of the cone, $R(x) = \alpha x$, where $\hat{\mathbf{x}}$ is the radial unit vector from the virtual origin and r is the distance from the axis. The applied electric field is axial, $\mathbf{E} = \hat{\mathbf{x}} E(x) = \hat{\mathbf{x}} E_0 (x_0/x)^2$. We consider the problem in the lubrication limit $\alpha \ll 1$. Since we are assuming that the electric field is independent of r, conductivity variations across the capillary must be negligible, which implies that we are also assuming small electric potential $e|\phi(x,r)|/kT \ll 1$, where $\phi(x,r)$ is the equilibrium potential due to the DNA and the charged capillary wall, e is the proton charge and kT the Boltzmann temperature. As we shall see, the velocity field is proportional to $E(x)$ and thus, $\propto 1/x^2$, so that continuity is satisfied at constant pressure. Thus, the fluid velocity $u(x,r)$ satisfies the constant pressure lubrication equation in cylindrical co-ordinates:

$$\mu \frac{1}{r} \left(r \frac{\partial u}{\partial r} \right) + \rho_e E(x) = 0 \tag{24}$$

where the charge density ρ_e is related to the equilibrium potential $\phi(x,r)$ as

$$-\epsilon \frac{1}{r} \left(r \frac{\partial \phi}{\partial r} \right) = \rho_e. \tag{25}$$

On eliminating ρ_e between Supplementary Equation (24) and Supplementary Equation (25) we find that $\bar{u} = u - (\epsilon E/\mu)\phi$ satisfies

$$\frac{1}{r} \left(r \frac{\partial \bar{u}}{\partial r} \right) = 0 \tag{26}$$

so that

$$u(x,r) - \frac{\epsilon}{\mu} E(x) \phi(x,r) = A(x) \ln r + B(x). \tag{27}$$

To determine $A(x)$ and $B(x)$ we assume that the potentials on the surface of the DNA (ζ_p) and the wall (ζ_s) are known and that the no-slip conditions are satisfied at fluid boundaries. Thus,

$$\phi(x,a) = \zeta_p, \quad \phi(x,R) = \zeta_s, \tag{28}$$

$$u(x,a) = v, \quad u(x,R) = 0. \tag{29}$$

The solution for the flow field is then

$$u(x,r) = \frac{\epsilon E(x)}{\mu} \left\{ \phi - \zeta_s - \Delta\zeta \, \frac{\ln(r/R)}{\ln(a/R)} \right\} + v \, \frac{\ln(r/R)}{\ln(a/R)}, \tag{30}$$

where $\Delta\zeta = \zeta_p - \zeta_s$. The viscous drag per unit length on the DNA may now be found:

$$\begin{aligned} f_v(x) &= 2\pi a\mu \left.\frac{\partial u}{\partial r}\right|_{r=a} \\ &= E(x)\left\{2\pi a\epsilon(\partial_r\phi)_{r=a} + 2\pi\epsilon\frac{\Delta\zeta}{\ln(R/a)}\right\} - \frac{2\pi\mu v}{\ln(R/a)} \end{aligned} \tag{31}$$

By Gauss's law first term in the bracket is $-\lambda_e$ and thus, the net force on the DNA per unit length is

$$\begin{aligned} f(x) &= f_v(x) + f_e(x) \\ &= 2\pi\epsilon\ \Delta\zeta\frac{E(x)}{\ln(\alpha x/a)} - 2\pi\mu v\frac{1}{\ln(\alpha x/a)}, \end{aligned} \tag{32}$$

where $f_e(x) = \lambda_e E(x)$ is the electric driving force. The requirement that the DNA moves force free is then

$$F = \int_{x_0}^{x_0+L} f(x)\, dx = -2\pi\mu v\int_{x_0}^{x_0+L}\frac{dx}{\ln(\alpha x/a)} - 2\pi\epsilon v_{e0}\int_{x_0}^{\infty}\frac{x_0^2}{x^2\ln(\alpha x/a)}\, dx = 0, \tag{33}$$

where we have replaced the upper limit of integration by infinity in the term with the rapidly decaying integrand and Supplementary Equation (12) readily follows from Supplementary Equation (33).

Note that these equations are valid provided the zeta-potentials are constant. If the problem is changed to one where, for example, the charge densities were constant then the zeta-potentials can be found using the solution of the Debye Hückel equation in an annulus. The zeta-potentials would then vary in the axial direction. In this case, axial pressure gradients are induced [6] and the above solution will no longer be strictly valid. However, if α is small, the correction to the flow due to the induced pressure gradient is also small and can be neglected at leading order.

Supplementary References

[1] Bell, N. A. W. & Keyser, U. F. Digitally encoded DNA nanostructures for multiplexed , single-molecule protein sensing with nanopores. *Nat. Nanotech.* **11**, 645–651 (2016).

[2] Bell, N. A. W. & Keyser, U. F. Specific Protein Detection Using Designed DNA Carriers and Nanopores. *JACS* **137**, 2035–2041 (2015).

[3] Kowalczyk, S. W., Wells, D. B., Aksimentiev, A. & Dekker, C. Slowing down DNA translocation through a nanopore in lithium chloride. *Nano Lett.* **12**, 1038–1044 (2012).

[4] Abramowitz, M. *Handbook of Mathematical Functions, With Formulas, Graphs, and Mathematical Tables,* (Dover Publications, Incorporated, 1974).

[5] Gere, J. & Timoshenko, S. *Mechanics of materials.* General Engineering Series (PWS Pub Co., 1997).

[6] Ghosal, S. Lubrication theory for electro-osmotic flow in a microfluidic channel of slowly varying cross-section and wall charge. *J. Fluid Mech.* **459**, 103–128 (2002).
